# Structural characterization of uranium and lanthanide loaded borosilicate glass matrix

**DOI:** 10.1038/s41598-025-13166-1

**Published:** 2025-08-04

**Authors:** I. Tolnai, J. Osan, P. Jovari, F. Pinakidou, A. Sulyok, M. Fabian

**Affiliations:** 1 Environmental Physics Department, HUN-REN Centre for Energy Research, Konkoly Thege St. 29-33, Budapest, 1121 Hungary; 2 HUN-REN Wigner Research Centre for Physics, Konkoly Thege St. 29-33, Budapest, 1121 Hungary; 3 School of Physics, Aristotle University of Thessaloniki, Thessaloniki, 54124 Greece

**Keywords:** Borosilicate glass, Neutron diffraction, Reverse monte carlo simulation, X-ray absorption spectroscopy, X-ray photoelectron spectroscopy, Leaching test, Condensed-matter physics, Structural materials, Techniques and instrumentation

## Abstract

Borosilicate glass is a potential candidate for high-level radioactive waste conditioning, thus understanding the effects caused by the combined presence of uranium and actinides within these matrices is of great importance. The glass matrix was simultaneously loaded with UO_3_ and lanthanide oxides (CeO_2_, Nd_2_O_3_, and Eu_2_O_3_) as chemical surrogates for actinides. Neutron diffraction in combination with Reverse Monte Carlo simulation confirmed that the basic glass structure is comprised of tetrahedral SiO_4_, and BO_3_/BO_4_ units. X-ray absorption spectroscopy indicated the presence of Ce mainly as Ce^III^ and the co-existence of U^V^ and U^VI^. U acts as an intermediate oxide and reduces the number of four-coordinated B, lanthanide ions serve as modifiers, with their increasing concentration shifting the B-O coordination from 3 to 4. X-ray photoelectron spectroscopy revealed a depth-dependent variation in the U^IV^/U^VI^ ratio. Leaching tests showed increased dissolution of Si, B, and Na, compared to the glass matrix.

**Supplementary Information:**

The online version contains supplementary material available at 10.1038/s41598-025-13166-1.

## Introduction

 Electricity generation via nuclear energy results in the production of spent nuclear fuel (SNF). This material requires careful handling, and the proper management of the resulting waste generally involves deep geological storage within an engineered barrier system (EBS). Prior to final disposal, the SNF is reprocessed to recover valuable elements, as it still contains up to ~ 94% U and ~ 1% Pu, which can be converted into Mixed Oxides for further reuse in the nuclear fuel cycle. In the remaining elements long-lived radioactive actinides, such as Th, Np, Am and Cm can be found^1^. Due to its significant radioactivity after reprocessing, it is categorized as high-level radioactive waste (HLW). Vitrification is a widely accepted method for immobilizing HLW, as the first barrier of EBS involving the encapsulation of HLW materials in a long-term, inert borosilicate glass matrix due to its excellent chemical and mechanical properties, thermal and radiation stability^2–4^. Therefore, it is crucial to investigate the effects of radioactive chemical constituents on the structural stability and solubility characteristics of the glassy system. In our previous studies we focused on the immobilization of lanthanide rare earth elements as minor actinide surrogates due to their similar chemical properties, including comparable ionic radii^5,6^. Ce^III^ was used to model Pu^III^, Nd^III^ was used to model Cm^III^, and Eu^III^ was used to model Am^III^. The glass matrix was loaded with three different lanthanide oxides at concentrations of 10 wt% and 30 wt%. Characterization aimed to explore how the incorporation of lanthanides (Ln): Ce, Nd, and Eu ions affect the atomic properties including bond lengths and coordination environments, and fundamental structure of the glass matrix^7^. Among the non-radioactive surrogates, Ce, introduced as cerium(IV) oxide, was present in both Ce^III^ and Ce^IV^ forms, as elevated sample preparation temperatures readily reduced it to the trivalent state. In another study, we investigated a series of borosilicate model waste glasses, where the matrix glass was loaded with UO_3_ at four different concentrations ranging from 10 to 40 wt% ^8^. To guarantee the long-term stability of the waste glass for containment, it was important to assess its physical, chemical properties and structural characteristics.

The focus of this study is on the structural changes that occur in the borosilicate glass when loaded with mixture of lanthanides and uranium – a novel combination that has not been explored yet. The simulated waste form incorporates cerium(IV) oxide, neodymium(III) oxide, europium(III) oxide, and uranium(VI) oxide, allowing a comprehensive analysis of the combined effects of these elements, thus providing a more realistic model for nuclear waste encapsulation. Four distinct glass samples were synthetized and investigated to gain deeper insight into the structural evolution of this complex system. The glass matrix was previously synthesized and characterized^9^. Neutron diffraction (ND) experiments, along with Reverse Monte Carlo (RMC) simulations were used to determine the structural parameters of the mixed (U, Ln) borosilicate glasses. X-ray absorption fine structure (XAFS) spectroscopy measurements were carried out at synchrotron radiation sources to better understand the local environment surrounding the three lanthanides and uranium. Given that the conditioned waste must be stored in deep underground repositories using an EBS approach, these glassy waste forms will serve as the initial containment barrier^10,11^. The long-term chemical durability of the borosilicate model waste glass series was assessed through water alteration experiments. Leaching tests were performed with porewater in corporation with thermal analysis techniques^12,13^. As Boda Claystone Formation (BCF) is the candidate host rock system for Hungarian HLW storage, the experiments utilized synthetic porewater formulated to replicate BCF characteristics. In addition to analyze the leachates for chemical composition, it is necessary to investigate the glass surface in contact with the liquid solution. To achieve this, we examined the glass surface using X-ray photoelectron spectroscopy (XPS).

This study investigates the structural properties of conditioned radioactive waste within a glass matrix, emphasizing how these characteristics change when incorporating both uranium and lanthanides, in contrast to matrices containing only uranium or only lanthanides, the durability of the glass matrix and the combined behavior of these elements in the matrix are also considered. A comparison of these new results with those from our previous studies and other applicable literature is discussed.

## Materials and methods

### Sample Preparation

The borosilicate glass system was synthesized using high-purity, analytical-grade oxide materials, including SiO_2_, Na_2_O, BaO, ZrO_2_, B_2_O_3_, CeO_2_, Nd_2_O_3_, and Eu_2_O_3_ sourced from Sigma-Aldrich in Budapest, Hungary, while UO_3_ was supplied by Reactivul, Bucharest, Romania. The isotopic enrichment of B_2_O_3_ focused on increasing the abundance of the ^11^B isotope to limit neutron absorption from ^10^B in natural boron. Through inductively coupled plasma mass spectrometry, the ^11^B isotope enrichment was determined to be 99.6%^14^. A high-temperature electrical furnace, along with a platinum crucible, was used under atmospheric conditions for sample preparation. Glasses were synthesized by melting the homogenized powder mixtures at 1450 °C and maintaining this temperature for 2 h. The molten material was then cooled to 1200 °C and quickly quenched on a stainless-steel plate. The bulk samples were powdered to 50 μm particle size through ball-milling with zirconia balls in a Retsch MM400 Mixer Mill. The following samples were synthetized and investigated: 70 wt% [Matrix] + 20 wt% UO_3_ + 10 wt% CeO_2_, 70 wt% [Matrix] + 20 wt% UO_3_ + 10 wt% Nd_2_O_3,_ 70 wt% [Matrix] + 20 wt% UO_3_ + 10 wt% Eu_2_O_3,_ and 60 wt% [Matrix] + 10 wt% UO_3_ + 10 wt% CeO_2_ + 10 wt% Nd_2_O_3_ + 10 wt% Eu_2_O_3,_ where the matrix composition was 55SiO_2_·10B_2_O_3_·25Na_2_O·5BaO·5ZrO_2_ (mol%), (denoted as MUCe, MUNd, MUEu and MUCNE, respectively and the composition of the matrix glass referred as REF). Glass density was determined gravimetrically at 22 ± 0.5 °C using an electronic balance with a 10⁻⁴ g sensitivity, with distilled water serving as the immersion medium. Each sample underwent four measurements, yielding a standard deviation of less than 0.01 g/cm^3^. The measured density of the glasses was 2.73 ± 0.01 g/cm^3^, 2.88 ± 0.01 g/cm^3^, 2.94 ± 0.01 g/cm^3^, 3.05 ± 0.01 g/cm^3^ and 3.18 ± 0.01 g/cm^3^ for REF, MUCe, MUNd, MUEu and MUCNE, respectively.

### X-ray absorption fine structure

Quick X-ray absorption spectrometry (QXAS) measurements were conducted at the P65 Applied XAFS beamline of the PETRA III synchrotron radiation source (DESY, Hamburg, Germany)^15^. Both X-ray absorption near-edge structure (XANES) and extended X-ray absorption fine structure (EXAFS) regions were covered by energy scans using a Si(111) monochromator around the L_III_ absorption edge of Eu and a Si(311) monochromator around the K-edges of Ce and Nd. The QXAS system enables a rapid acquisition of absorption spectra with a step scan time of 0.1 s and energy step of 0.5 eV.

XAFS measurements were also conducted at the B18 XAS beamline of the Diamond Light Source (Harwell, United Kingdom)^16^. Spectra were acquired in QXAS mode utilizing a Si(111) monochromator around the L_III_ absorption edges, covering XANES regions of Ce, Nd and Eu and both XANES and EXAFS regions for U. The experiment employed a step scan time of 0.06 s and an energy step of 0.3 eV. XAFS spectra were collected at both beamlines in transmission mode using ionization chambers for monitoring incoming and transmitted X-ray intensities. The glass samples, pressed into 13 mm diameter pellets with polyvinylpyrrolidone as the binder, were examined. The optimal quantities used during sample preparation were calculated using the XAFSmass program^17^. Similarly prepared pressed pellets from UO_2_, UO_3_, CeO_2_, Nd_2_O_3_, and Eu_2_O_3_ were measured as reference compounds. All spectra were collected in ambient conditions. Data processing, including background removal, XAFS spectra normalization and linear combination fitting (LCF) was carried out with the Athena software package^18^. EXAFS analysis utilized FEFF8.2 to calculate theoretical phase and amplitude functions for the scattering paths, while curve fitting was conducted in both R-space and k-space with the FEFFIT tool^19^.

### Neutron diffraction

For the MUEu and MUCNE glassy samples, ND measurements were carried out using the PSD diffractometer (*λ*_0_ = 1.068 Å, *Q* ≤ 9.8 Å^−1^)^20^ at the Budapest Neutron Centre, while the MUCe and MUNd samples were measured with the 7C2 diffractometer (*λ*_0_ = 0.726 Å, *Q* ≤ 15.3 Å^−1^)^21^ at the Laboratoire Léon Brillouin. Each powder sample, weighing about 5 g, was placed in thin-walled cylindrical vanadium sample holders with diameters of 8 mm (PSD) and 6 mm (7C2). The raw data were corrected for detector efficiency, background scattering, and absorption effects. Total structure factors were calculated using local software packages, and a detailed description of the data evaluation was provided previously^9^.

### Reverse Monte Carlo simulation

The RMC simulation is a powerful method for constructing large 3D structural models compatible with experimental data, particularly S(*Q*) diffraction structure factors^22^ and *χ(k)* EXAFS curves^23^ and various physical and chemical constraints (density, minimum interatomic distances, coordination numbers, bond angle distributions or even Q^n^ distributions). In this paper structural models are generated by fitting neutron diffraction structure factors and EXAFS data measured at the U L_III_-edge. Simulations are carried out by the RMC + + code^24^. The RMC algorithm calculates the one-dimensional partial atomic pair correlation functions *g*_ij_(*r*), and then uses inverse Fourier transformation to derive the partial structure factors *S*_ij_(*Q*), as described in Eq. (1):

1 $$\:{S}_{ij}\left(Q\right)=1+\frac{4\pi\:{\rho\:}_{0}}{Q}\int\:r\left[{g}_{ij}\left(r\right)-1\right]\text{s}\text{i}\text{n}Qr\:\text{d}r$$

where *ρ*_0_ denotes the atomic number density. The simulation process involves adjusting the atomic positions randomly until the calculated *S*(*Q*) matches the experimental data within the experimental error margin.

Using the RMC method, *g*_*ij*_(*r*) were obtained. The weighting factors, *w*_*ij*_ and *S*_*ij*_(*Q*) were defined by Eq. (2) and Eq. (3):

2 $$\:{w}_{ij}=\frac{{c}_{i}{c}_{j}{b}_{i}{b}_{j}}{{\left[\sum\:_{i,j}^{k}{c}_{i}{b}_{j}\right]}^{2}}$$

3 $$\:S\left(Q\right)=\sum\:_{i,j}^{k}{w}_{ij}{S}_{ij}\left(Q\right)$$

where *c*_*i*_ and *c*_*j*_ are the molar fractions of the components, *b*_*i*_ and *b*_*j*_ are the coherent neutron scattering lengths, and *Q* stands the momentum transfer. In the case of MUCe, MUNd and MUEu *k* = 8 (number of different components in the sample) which corresponds to a total of 36 atom pairs based on *k*(*k* + 1)/2 = 36 and in the case of MUCNE sample the total atom pairs is 55. The neutron-weighted factors for the atomic pairs with the largest contribution to the ND can be found in Table 1.

**Table 1:** Neutron-weighted factors, *w*_*ij*_ [%], for the *X*-O and O-O partial interatomic correlations.

	Weighting factor, w_ij_ [%]									
	Si-O	B-O	Na-O	Ba-O	Zr-O	Ce-O	Nd-O	Eu-O	U-O	O-O
REF	18.68	10.32	12.24	1.58	3.68	-	-	-	-	39.95
MUCe	15.68	7.27	12.47	1.65	2.46	4.03	-	-	1.91	42.30
MUNd	15.42	7.16	12.27	1.65	2.45	-	3.96	-	3.12	41.04
MUEu	15.47	7.20	12.33	1.70	2.43	-	-	4.00	2.79	41.32
MUCNE	14.51	6.79	11.65	1.62	2.32	1.82	1.80	2.88	2.74	40.80

Model *χ*(*k*) EXAFS curves are obtained from the partial correlation functions by the following Eq. (2)^3^:

4 $$\:{\chi\:}_{i}\left(k\right)=\sum\:_{j}{\int\:}_{0}^{{r}_{max}}4\pi\:{\rho\:}_{0}{c}_{j}\:{r}^{2}{g}_{ij}\left(r\right){B}_{ij}(k,r)\text{d}r$$

where *i* denotes the absorbing component, *j* runs over all components, while *B*_*ij*_(*k*,*r*) is the ‘backscattering matrix’ that gives the *k*- and *r*-dependent elementary contribution of a *j* type neighbor to the model curve. The right hand side of Eq. (4) is the superposition of backscattering contributions from all neighboring atoms.

As backscattering of photoelectrons is strongly damped by distance, EXAFS spectra of glasses usually contain information only on the first coordination shell. (It is to be noted that for crystalline systems the contribution of further shells should often be taken into account^25^. It was assumed that, in the glasses investigated, only oxygen atoms can be found in the first coordination shell of U atoms. Therefore, model U L_III_ EXAFS spectra depend directly only on U-O correlations. Elements of the U-O backscattering matrix were calculated by the FEFF9 code^26^.

Starting configurations were obtained by placing 10,000 atoms randomly in cubic boxes. The sizes of the simulation boxes were chosen to comply with the experimentally determined density (see above). The RMC simulations incorporated two types of constraints: minimum interatomic distances (cut-offs) and two coordination constrains. Si-O and B-O cut-offs were 1.4 Å and 1.2 Å, respectively. Minimum metal-O distances were 1.7–1.8 Å, while all metal-metal cut-offs were 3 Å. Minimum Si-Si, Si-B and B-B distances were chosen to be 2.8 Å, 2.5 Å and 2.3 Å, respectively The cut-off values were based on earlier results for SiO_2_-Na_2_O^27^ B_2_O_3_-Na_2_O glasses^28^ the matrix glass^9^ the matrix glass with lanthanides^29^ and the matrix glass loaded with UO_3_^8^.

Coordination constraints can help to reduce the uncertainty of the structural parameters deduced from RMC-generated models. In the present study the following coordination constraints were applied for all glasses investigated: (i) the Si-O coordination number was constrained to be 4 for all Si atoms, (ii) each B atom had to have at least 3 O neighbors (3 and 4 were both allowed). The above coordination constraints were satisfied usually at least by 88% and 96% of the atoms for the Si-O and B-O, respectively. In addition, the average U-O coordination number was also constrained to the values obtained by EXAFS data analysis (see below).

### X-ray photoelectron spectroscopy

XPS was used to determine sample composition at the surface and in the surface range using an ESCALAB Xi^+^ equipment from Thermo Scientific. Three positions were detected on each mm-sized glass piece. Due to the insulating nature of the samples, continuous dual charge compensation (both positive and negative) was applied to prevent electrical charge-up. However, the curvature of the glass samples caused uneven charging, leading to a widening of the detected XPS peaks. The detected peaks included the main components Si 2p (99 eV) and O 1 s (532 eV), along with dopants such as B 1 s (186 eV), Ba 3 d (780 eV), Zr 3 d (182 eV), Na 1 s (1072 eV), C 1 s (284 eV), Ce 3 d (882 eV), Nd 3 d (982 eV), Eu 3 d 5/2 (1134 eV), and U 4f 7/2 (380 eV). Observations took place analyzing the sample surface in “as received” state that involves some organic contamination. Effort to remove surface contamination (e.g. Ar cluster sputtering) was not successful since the sputtering caused changes in the matrix itself too. On the MUE and MUCNE samples depth profile measurements were carried out with Ar^+^ ion sputtering to reveal the depth dependence of composition. Ion sputtering was performed using a 500 eV Ar^+^ beam at a 45° angle of incidence, scanned across a 2 mm × 2 mm area. This process removed approximately 2 nm of material per step, with a total removal of about 30 nm. The XPS spot size for measurement was 500 μm, focused at the center of the sputtered square. The measured spectra were evaluated by determining the peak intensities. A peak fitting algorithm was applied to decompose complex peak shapes and separate the Na Auger peak from the oxygen 1 s peak. Component concentrations were calculated using sensitivity factors from the ALTHERMO1 library, assuming a homogeneous target.

### Leaching test

The Product Consistency Test is an effective method for assessing the chemical durability of glassy wasteform systems and the alterations in their structure resulting from interactions with leaching solutions. This test provides valuable data on both the release rates of structurally immobilized uranium and actinide surrogates and the alteration in the composition of the leaching solution. According to the ASTM C1285-21 protocol^30^ the normalized release rates and glass dissolution rates for silicate, boron, uranium, and lanthanides can be determined using Eq. (5) and Eq. (6):

5 $$\:{NL}_{i}=\frac{{c}_{i}\left(sample\right)}{\left({f}_{i}\right)\bullet\:\left(\frac{SA}{V}\right)}$$

6 $$\:{NR}_{i}=\frac{{c}_{i}\left(sample\right)}{\left({f}_{i}\right)\bullet\:\left(\frac{SA}{V}\right)\bullet\:\left(t\right)}$$

where *c*_*i*_ denotes the concentration of the element of interest in the soaking water (g/L), *f*_*i*_ is the weight fraction of the element in the original borosilicate glass (unitless), *SA/V* is the ratio of the surface area of the final waste form to the volume of the leachate (m^−1^) and *t* indicates the duration of the test (days).

 Since the BCF is regarded as a potential host rock system for HLW storage in Hungary, experiments were conducted using synthetic porewater designed to reflect the characteristics of the albitic claystone in BCF. The porewater chemistry was modeled using the MINSORB geochemical speciation software and the Nagra/PSI 01/01 thermodynamic database^12,13^. The porewater composition was calculated with a fixed p_CO2_ of 10^−3.5^ bar and a pH of 8.0, maintaining equilibrium with atmospheric CO_2_ and saturation with calcite, dolomite, and quartz. To achieve charge neutrality, Na^+^ and Cl^−^ concentrations were adjusted, while concentrations of Ca, Mg, Si, and C(IV) were set according to predefined conditions^31^. The composition of the resulting synthetic Boda porewater (SBPW) is given in Table 2^32^.

**Table 2:** Chemical composition of the synthetic Boda Porewater (SBPW).

Element, ion	Concentration (mol/L)
Na	1.7 × 10^−2^
K	1.8 × 10^−4^
Mg	2.3 × 10^−3^
Ca	3.1 × 10^−3^
Sr	1.5 × 10^−5^
Cl^−^	2.3 × 10^−2^
SO_4_^2−^	1.9 × 10^−3^
HCO_3_/CO_3_	6.1 × 10^−4^
Ionic strength (mol/L)	3.3 × 10^−2^
pH	8.1
Eh (mV)	−300

For the test method, a 100 to 200 mesh fraction of the crushed sample is used, from which the adhering fine is removed. Around 1 g of the sample is placed into cylindrical 304 L stainless steel vessel, an amount of SBPW equal to ten times the sample mass is added. The sealed system is kept in a laboratory oven at a constant temperature of 90 ± 2 °C. The surface area to volume ratio (*SA/V*) was determined to be 1600 m^−1^ for all samples, based on the assumption that the glass particles were cubic in shape throughout the calculations. After 3, 7, and 10 days, the vessels were unsealed, and the leachates were passed through a 0.45 μm syringe filter. Following filtration, the leachates were acidified with concentrated HNO_3_ and analyzed by inductively coupled plasma optical emission spectroscopy (ICP-OES) using a Perkin Elmer Avio 200 ICP-OES instrument.

## Results and discussion

### Local environments of uranium and lanthanides with EXAFS

**Fig. 1:**
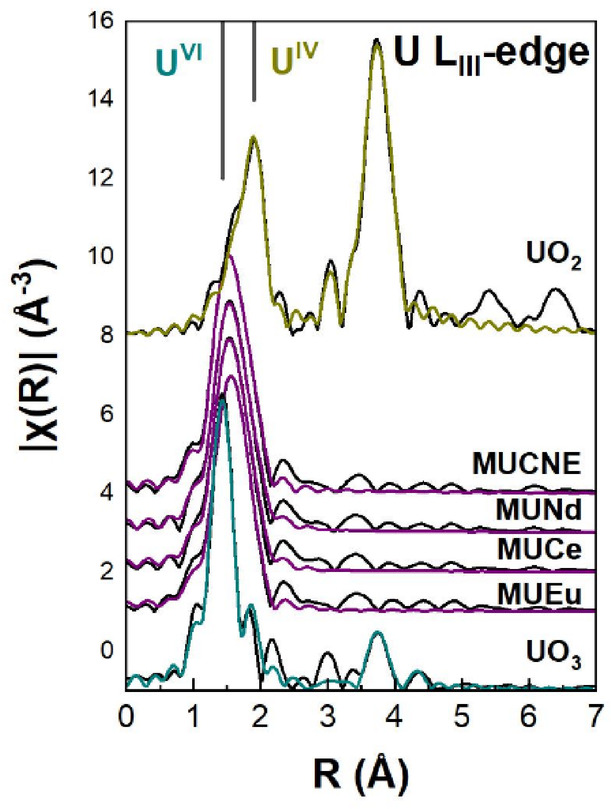
Fourier transforms of the *χ*(*k*) U L_III_ EXAFS spectra for the Ln and U loaded glassy samples. The experimental data are shown in black while the fitting is depicted with the color line. The figure also includes experimental data and fitting curve of reference UO_3_ and UO_2_.

The U L_III_-edge EXAFS spectra were fitted assuming the presence of U^VI^ ions as uranyl in a linear arrangement with 2 O atoms (O = U = O) and 6 O atoms in an equatorial plane and U^V^ in octahedral coordination, denoted as U^VI^-O_axial_, U^VI^-O_equatorial_ and U^V^-O in Table 3 respectively. In particular, the fitting of the χ(*k*) EXAFS spectra was performed simultaneously for all studied samples within the *k*-range of 3–13 Å^−1^. Curve fitting was performed in the first nearest neighbor (nn) shell, with the fitting parameters including the percentage of U atoms in the two different sites, the Debye-Waller (*DW*) factors and the U-O bond length. The values of the amplitude reduction factor (*S*_*0*_^*2*^) and energy origin (*E*_*0*_) were constrained to be equal although iterated in order to provide the best fitting results. The Fourier Transforms (FTs) of the *χ*(*k*) EXAFS spectra are presented in Fig. 1, the raw and fitted χ(*k*) EXAFS are shown in Figure S1(a) in the Supplementary material, while the best fit parameters are included in Table 3. Compared to the previous set of glass samples with U, a notable finding is the slight increase of the U^V^-O bond length compared to the MU10-MU40 glasses^8^ by 0.05–0.07 Å. This change is accompanied by an increase in the static disorder of the U^V^O_5_ octahedra, as the increased values of the *DW* factors (~ 15 × 10^−3^ Å^2^ suggest. However, the calculated U-O bond lengths are consistent with those found in comparable glassy samples^3,33^ and shows a high level of consistency with the results derived from RMC.

**Table 3:** Best fit parameters obtained from the U-L_III_ EXAFS fitting results of the Ln and U loaded glass samples.

	U^VI^-O_axial_				U^VI^-O_equatorial_			U^V^-O			
	*N*(O)	U^VI^ [at%] (x)	*R* [Å]	DW [×10^−3^ Å^2^]	*N*(O)	*R* [Å]	DW [×10^−3^ Å^2^]	*N*(O)	U^V^ [at%] (1-x)	*R* [Å]	DW[×10^−3^ Å^2^]
MUCe	2 × *x*	75 ± 6	1.85 ± 0.01	1.5	6 × *x*	2.30 ± 0.03	14.8	6 × (1-*x*)	25 ± 6	2.20 ± 0.02	2.4
MUNd	2 × *x*	65 ± 8	1.83 ± 0.01	1.5	6 × *x*	2.29 ± 0.03	13.6	6 × (1-*x*)	35 ± 8	2.20 ± 0.02	3.1
MUEu	2 × *x*	63 ± 6	1.85 ± 0.01	2.3	6 × *x*	2.28 ± 0.03	13.2	6 × (1-*x*)	37 ± 6	2.18 ± 0.02	1.7
MUCNE	2 × *x*	73 ± 7	1.84 ± 0.01	1.4	6 × *x*	2.28 ± 0.03	13.8	6 × (1-*x*)	27 ± 7	2.20 ± 0.02	1.5

As it is revealed by the EXAFS analysis results, in our sample series a weak, systematic decline in the atomic percentage of hexavalent uranium in the glass can be observed when lanthanides with higher atomic numbers (*Z*) are used. This trend is shown in Figure S2 in Supplementary material.

Sample thickness was optimized specifically for the U L_III_-edge, with adjustments limited by regulatory restrictions on U handling. Since optimal Ce, Nd, and Eu measurements required thicker samples, supplementary samples without U were prepared at these increased thicknesses for optimal EXAFS data acquisition including one sample containing all relevant lanthanides: 70 wt% [Matrix] + 10 wt% CeO_2_ + 10 wt% Nd_2_O_3_ + 10 wt% Eu_2_O_3_ (MCeNdEu) and two others with CeO_2_ added at different concentrations: 90 wt% [Matrix] + 10 wt% CeO_2_ (MCe10) and 70 wt% [Matrix] + 30 wt% CeO_2_ (MCe30). These CeO_2_ loaded samples are directly comparable to those used in our previous study^7^.

**Fig. 2:**
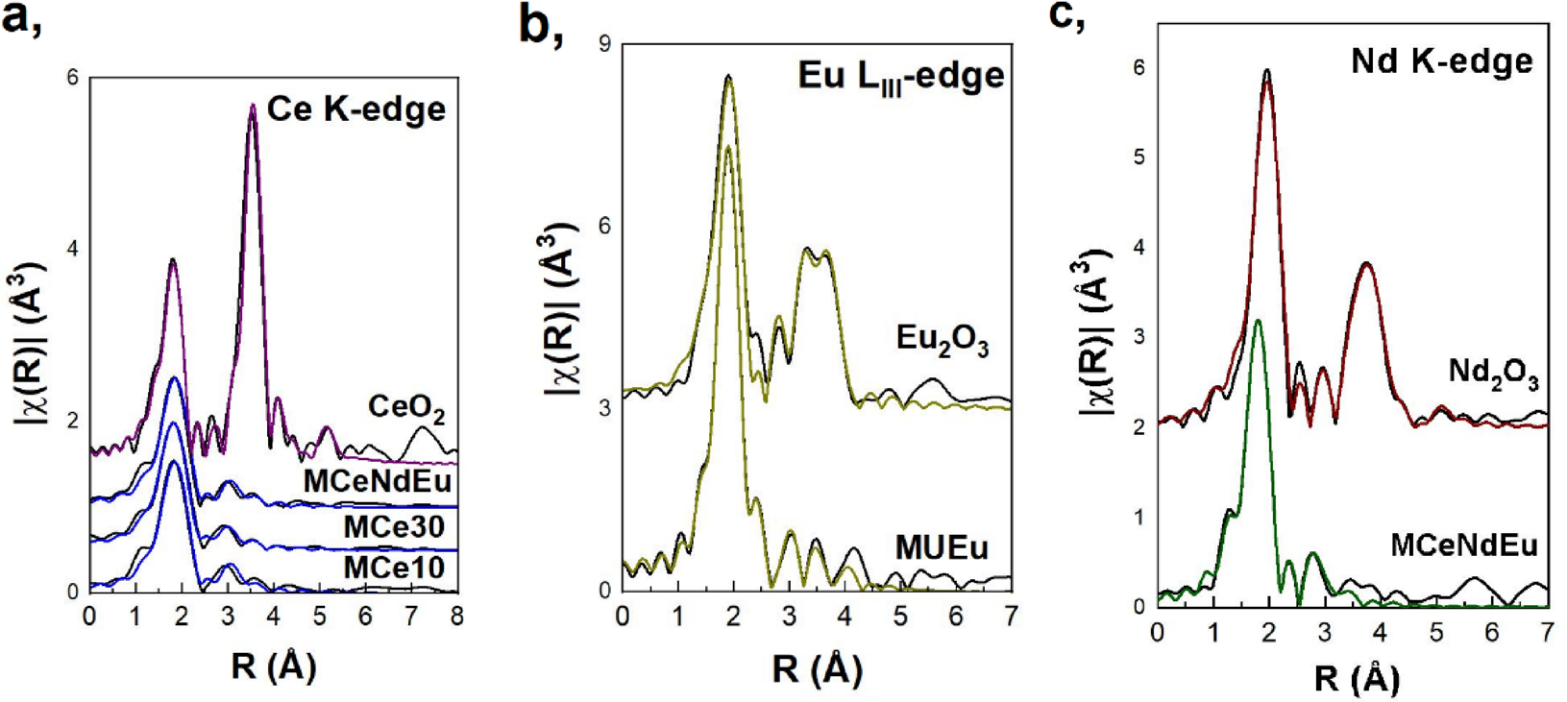
Fourier Transforms of the *χ*(*k*) EXAFS spectra for the supplementary Ce loaded samples and MCeNdEu recorded at the Ce K-edge (**a**), for the MUEu sample at the Eu L_III_-edge (**b**), and for the MCeNdEu sample at the Nd K-edge (**c**). The experimental data and fitting curve of reference CeO_2_, Eu_2_O_3_ and Nd_2_O_3_ are also included.

The Ce K-edge EXAFS spectra of the MCe10, MCe30 and MCeNdEu glasses were fitted assuming that Ce atoms form octahedra which link to SiO_4_ polyhedra in the matrix. Curve fitting proceeded in the two nn shell (*k*-range: 3-11.5 Å^−1^)and the fitting parameters were the coordination numbers, *DW* factors and atomic distances in all shells. The values of the *S*_*0*_^*2*^ and *E*_*0*_ were constrained to be equal although iterated. The FTs of the experimental data and the fitting are shown in Fig. 2(a). The respective raw and fitted *χ*(*k*) EXAFS are shown in Figure S1(b) in the Supplementary material. The fitting results, listed in Table 4, indicate the presence of Ce in octahedral coordination. These results are in good agreement with the RMC calculations according to which the O atoms are expected at a distance of 2.55 Å from the Ce atoms^29^. Furthermore, both the coordination number and Ce-O bond length suggest the network forming role of Ce, contrary to CeO_2_-silicate glasses where Ce acts as a glass former^34^. Additionally, the Ce-O bond length is much longer than the respective in the Ce^IV^ reference oxide, an indication of a change in the valence state of Ce (reduction of Ce^IV^)^35^.

**Table 4:** Best fit parameters obtained from the Ce-K EXAFS results of the Ln loaded glassy samples. Values in bold were kept fixed throughout the fitting process.

	*N* (O)	*R*_Ce−O_ [Å]	DW [×10^−3^ Å^2^]	*N* (Si)	*R*_Ce−Si_ [Å]	DW [×10^−3^ Å^2^]
MCe10	6.0 ± 0.4	2.44 ± 0.01	14.9	1.5 ± 0.3	3.67 ± 0.02	8.2
MCe30	5.9 ± 0.3	2.42 ± 0.01	15.7	0.9 ± 0.2	3.64 ± 0.02	5.7
MCeNdEu	5.5 ± 0.6	2.44 ± 0.01	13.8	1.0 ± 0.2	3.67 ± 0.02	4.2
CeO_2_	**8**	2.35 ± 0.01	**12**	12 (Ce)	3.85 ± 0.02	4.3

The Eu L_III_-edge spectrum of the U-rich sample MUEu was fitted assuming a mixed bonding environment of Eu in the borosilicate glass as shown in Fig. 2(b). The fitting of the *χ*(*k*) EXAFS spectra (as shown in Figure S1(c) in the Supplementary material) was performed in the *k*-range of 2.8–12.0 Å^−1^ in the two nn shells. The model used for the fitting assumes that a fraction *x* of Eu atoms is 10-fold coordinated with O atoms, surrounded by BO_4_ tetrahedra, based on the crystalline structure of LaB_3_O_6_, where La has been substituted with Eu^36^. The remaining *y =* 100 *– x* Eu atoms form pentagonal bipyramids EuO_7_ that connect via corners to SiO_4_ units^37^. The fitting parameters were the percentage of the Eu in the two distinct sites, the *DW* factors and the atomic distances in all shells and also the value of the *E*_*0*_. The value of the *S*_*0*_^*2*^ was kept fixed to the respective value derived from the analysis of the reference Eu_2_O_3_ (*S*_*0*_^*2*^ = 0.9). The best fit parameters are presented in Table 5.

**Table 5:** Structural parameters obtained from the Eu L_III_ EXAFS fitting results of the glassy samples. Values in bold were kept fixed throughout the fitting process.

Eu L_III_-edge			
	*N*	*R* [Å]	DW [×10^−3^ Å^2^]
MUEu		*x* = 79 ± 8 [at%]	
O	**10 ×** ***x***	2.41 ± 0.01	21.4
B	**10 ×** ***x***	3.37 ± 0.02	1.6
O	**7 × (1-** ***x*** **)**	2.34 ± 0.03	19.9
Si	**4 × (1-** ***x*** **)**	3.57 ± 0.04	7.1
Eu_2_O_3_			
O	**6**	2.37 ± 0.01	10.8
Eu	**6**	2.64 ± 0.02	8.5
Eu	**6**	3.82 ± 0.03	13.0
O	**8**	4.30 ± 0.04	5.8

No major changes were detected in the bonding environment of Eu as a result of U-incorporation, a finding that confirms that Eu acts as a typical network modifier, as previously revealed in the Matrix-Eu glasses^7^.

As in the case of Eu-glass, the Nd K-edge spectrum of the U-free glass MCeNdEu was fitted assuming a mixed bonding environment of Nd in the borosilicate glasses as presented in Fig. 2(c). The fitting of the *χ*(*k*) EXAFS spectrum (shown in Figure S1(d) in the Supplementary material) was performed in the *k*-range of 3.0–11.0 Å^−1^ in the two nn shells. In particular, it was assumed that a fraction *x* of Nd atoms is 10-fold coordinated to O and links to borate chains made up from [B_6_O_12_]_n_^6−^ structural units (the crystalline structure of EuB_3_O_6_ was used, where Eu has been substituted with Nd)^38^. The remaining *y* = 100 – *x* Nd atoms is 8-fold coordinated and link to SiO_4_ units^39^. The fitting parameters were the percentage of Nd in the two distinct sites, the *DW* factors and the atomic distances in all shells and also the value of the *E*_*0*_. The value of the *S*_*0*_^*2*^ was kept fixed to the respective value derived by the analysis of the reference N_2_dO_3_ (*S*_*0*_^*2*^ = 0.97). The corresponding fitting results are presented in Table 6.

**Table 6:** Best fit parameters obtained from the analysis of the Nd-Kedge EXAFS spectra. Values in bold were kept fixed throughout the fitting process.

Nd K-edge			
	*N*	*R* [Å]	DW [×10^−3^ Å^2^]
MCeNdEu		*x* = 76 ± 4.0 [at%]	
O	**10 ×** ***x***	2.41 ± 0.01	11.8
B	**10 ×** ***x***	3.38 ± 0.02	17.2
O	**8 × (1-** ***x*** **)**	2.35 ± 0.03	3.3
Si	**6 × (1-** ***x*** **)**	3.58 ± 0.04	6.3
Nd_2_O_3_			
O	**4**	2.40 ± 0.01	2.7
O	**4**	2.59 ± 0.02	2.8
Nd	**6**	3.67 ± 0.03	22.1
Nd	**6**	3.80 ± 0.04	9.8
O	**12**	4.45 ± 0.05	2.2
O	**9**	5.28 ± 0.06	16.3
Nd	**6**	5.33 ± 0.07	13.5

The fitting results are similar to the previously reported results on the MNd10 and MNd30 glasses. The environment of Nd is mainly borate, yet, both the B and Si atoms are located at a longer distance from Nd, i.e. approximately by 0.2 Å. Thus, the presence of U and Ce and Eu does not have any influence on the structural role of Nd in the glasses.

### Oxidation States of uranium and lanthanides with XANES

**Fig. 3:**
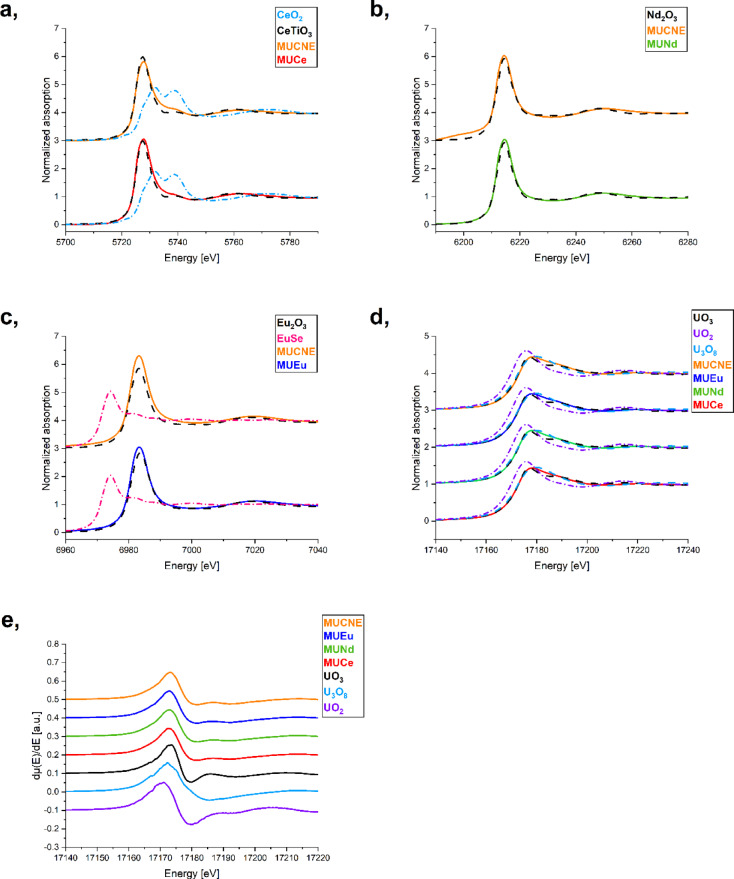
Ce L_III_ (**a**), Nd L_III_ (**b**), Eu L_III_ (**c**), and U L_III_ (**d**) XANES spectra of the glassy specimens compared to the XANES spectra of the reference compounds. The solid lines represent the spectra of the U and Ln loaded glass samples, while the dashed lines represent the spectra of the references. Additionally, the first derivates of the normalized XANES spectra (**e**) collected in transmission mode for the U-loaded samples and reference compounds.

The normalized XANES spectra collected at Ln and U L_III_-edges for glass samples and reference compounds are shown in Fig. 3. Based on our previous measurement results^29^ Ce is the only lanthanide expected to exhibit both III and IV oxidation states. During the glass synthesis, Ce was introduced into the system as CeO_2_. However, due to the high temperature during melt quenching, partial reduction of Ce^IV^ occurred. To determine the oxidation state of Ce, the normalized XANES spectra at the Ce L_III_-edge were compared to two reference spectra: CeO_2_ for the IV oxidation state and CeTiO_3_ for the III oxidation state. The results obtained from linear combination fitting (LCF) indicates that the experimental glass spectra align well with the Ce^III^ reference spectrum, which suggest an almost complete reduction of Ce in the glass samples, these results correlate well with those reported in^5^.

It was observed that the solubility of Ce in borosilicate glasses increases with temperature. At 1400 °C, the Ce^III^/Ce_total_ fraction reaches 0.9, indicating that Ce is almost completely reduced during the glass synthesis. In the MUCe sample, LCF analysis indicated 3.9% Ce^IV^ content, while in the MUCNE sample, 12.3% of Ce^IV^ was detected. These results were achieved with 10 wt% CeO_2_ loading, and it is suggested that the solubility could be increased to 13 wt% due to the reduction of Ce during the process^5^. This ratio obtained is higher than that achieved in our previous study, where only CeO_2_ was used for loading at varying concentrations^29^. As reported in literature^40^ Nd is well soluble in glass at concentrations below 4 mol%, resulting in an amorphous glassy system. In both the MUNd and MUCNE samples, Nd was found exclusively in the III oxidation state. The same observation applies to samples containing Eu, where both samples contain only Eu^III^ ions, despite the known redox sensitivity of europium. According to the findings of Cicconi, the Eu XANES spectra exhibit systematic variations with changes in glass composition^41^. For Eu doped granitic and basaltic silicate glasses, the Eu^II^/Eu_total_ molar ratio ranges from less than 1–22%, depending on the specific composition. In contrast, our previous analysis of the Eu loaded borosilicate glass indicate that Eu^II^ comprises less than 1% of the total Eu^7^. In the current U-containing samples, no Eu^II^ contribution was identified by LCF. However, considering the already very low proportion of Eu^II^ in the U-free glass, this absence is likely due to the limitations of the LCF method, rather than a specific effect of U. Reference spectra of Eu_2_O_3_ and EuSe were used for Eu^III^ and Eu^II^, respectively, in the LCF analysis.

During the analysis of the L_III_-edge spectra, and based on our prior results, we assumed that uranium introduced as U^VI^ may undergo reduction and be present in the U^V^ oxidation state, with the potential for U^IV^ as well. Accordingly, reference spectra for UO_3_, UO_2_, and U_3_O_8_ were used in LCF. The analysis revealed no indication of U^IV^, with the samples consistently showing the presence of U^VI^ and U^V^ in similar proportions, as detailed in Table 7. The partial reduction of uranium from U^VI^ to U^V^ can be explained by the high melting temperature used during glass preparation. It is known that elevated temperatures (1100–1450 °C) can shift the redox balance and lead to the formation of reduced uranium species, even without added reductants^3,42^. The presence of U^V^ is consistent with these conditions.

**Table 7:** Ratio of U^VI^ to U^V^ in the samples determined with LCF fitting, using UO_3_ as the reference for U^VI^, UO_2_ for U^IV^, and U^VI^U^V^_2_O_8_ for a mixture of 1/3 U^VI^ and 2/3 U^V^. The fitting range was set between − 20 and 70 ev from the absorption edge energy^43^.

	U^VI^ [%]	U^V^ [%]
MUCe	68 ± 2	32 ± 2
MUNd	65 ± 2	35 ± 2
MUEu	65 ± 2	35 ± 2
MUCNE	68 ± 3	32 ± 3

The results align closely with those reported in literature^33,43^ where typical ratios of 70% U^VI^ and 30% U^V^ were also observed. When compared to earlier samples loaded solely with U, it can be concluded that the presence of lanthanides does not influence the oxidation state of U^8^.

### Atomic structure from ND and U L_III_-edge EXAFS data via reverse Monte Carlo simulation

**Fig. 4:**
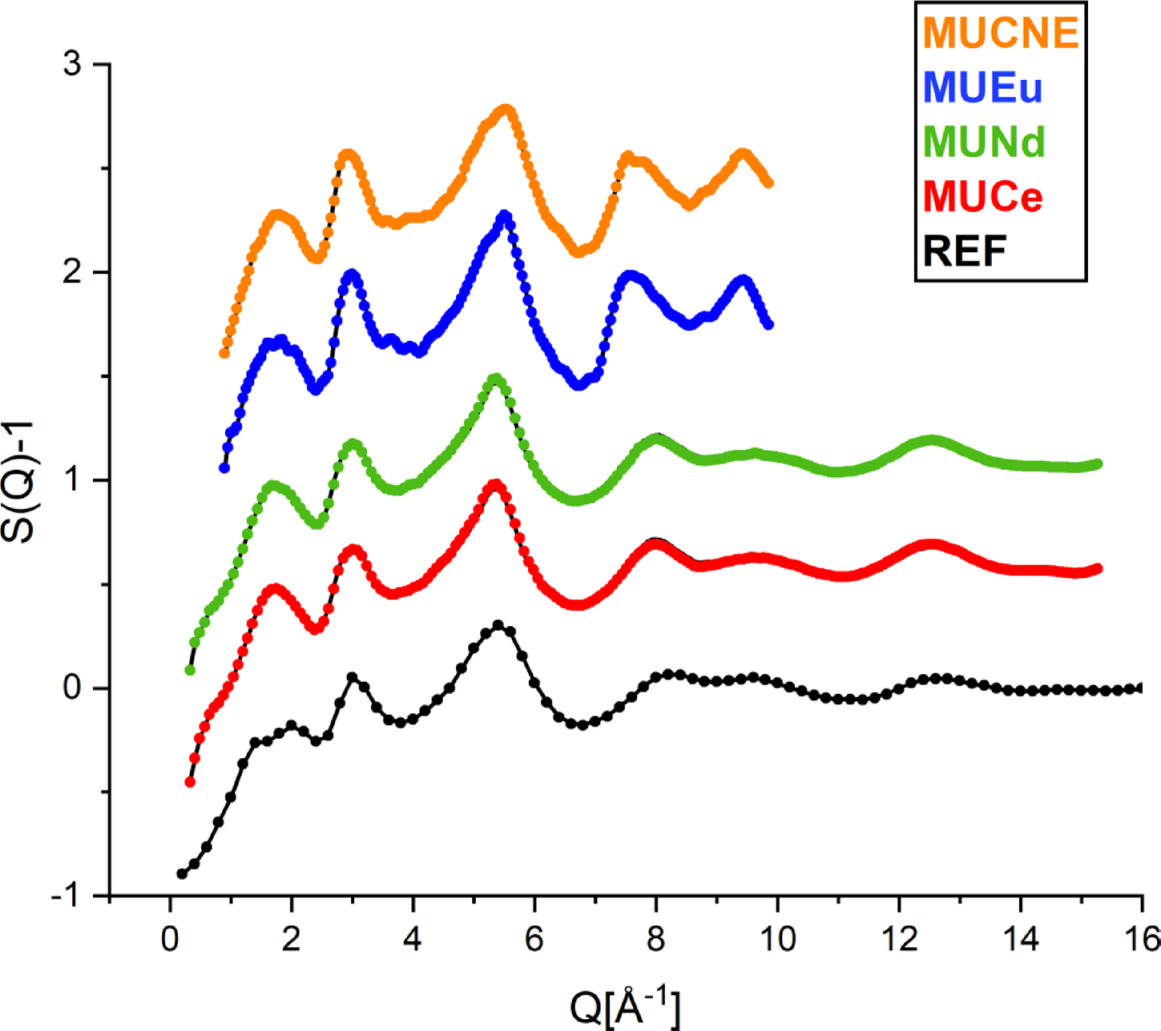
Total structure factors derived from ND on REF (black circles (7C2)), MUCe (red circles (7C2)), MUNd (green circles (7C2)), MUEu (blue circles (PSD)) and MUCNE (orange circles (PSD)), each data set is accompanied with the respective RMC fit curves (black solid lines).

Figure 4 presents the structure factors, *S*(*Q*) obtained from ND experiments alongside the *S*(*Q*) functions derived from RMC simulations.

The ND patterns reveal that the samples were fully amorphous, indicating that the implemented quenching was sufficient to ensure vitrification. The RMC calculations show excellent convergence, with the final simulated *S*(*Q*) closely matching the experimental data. Additionally, the ND experimental curves are quite similar across all samples, with only minor differences observed in the low *Q*-range around 1.4-2.0 Å^−1^ and between 9.0 and 10.0 Å^−1^. The basic structure of the (U, Ln) mixed glass remained highly similar to the REF glass, the peaks are observed at 2.9-3.0 Å^−1^ and 5.4–5.5 Å^−1^. Utilizing the RMC + + code, partial atomic pair correlation functions and their coordination numbers were generated. In principle, information on the coordination environments of the components can be obtained from the partial pair distribution functions produced by the RMC calculations. It should be noted, however, that due to the limitations of the experimental data and RMC the separation of all 36 partial pair correlation functions of an eight-component glass is not realistic and therefore beyond the scope of the present study. Therefore, we concentrate on the partial correlations that either have a large weight in the neutron diffraction or EXAFS datasets (Si-O, B-O, O-O, U-O) or pinned by the constraints applied (Si-Si). The corresponding distances including the second neighbor Si-Si and Si-B distances are shown in Table 8, while the coordination numbers are given in Table 9.

**Table 8:** Interatomic distances, *r*_*ij*_ [Å] obtained from the RMC simulations. Based on the reproducibility of the RMC runs, the estimated errors are also given.

	Interatomic distances, *r*_ij_ [Å]				
	Si-O	B-O	U-O	Si-Si	Si-B
REF ^9^	1.60 ± 0.005	1.40/1.60 ± 0.01	-	3.00 ± 0.1	2.5−3.1 (± 0.1)
MUCe	1.60 ± 0.02	1.34/1.59 ± 0.03	1.83/2.31 ± 0.02	3.10 ± 0.05	2.89 ± 0.05
MUNd	1.60 ± 0.02	1.33/1.59 ± 0.03	1.83/2.30 ± 0.02	3.10 ± 0.05	2.91 ± 0.05
MUEu	1.60 ± 0.02	1.54 ± 0.04	1.83/2.31 ± 0.02	3.06 ± 0.05	2.91 ± 0.05
MUCNE	1.60 ± 0.02	1.52 ± 0.04	1.83/2.32 ± 0.02	3.07 ± 0.05	2.85 ± 0.05

**Table 9:** Average coordination numbers, *CN*_*ij*_, determined from RMC simulations for Si-O, B-O, U-O and Ln-O atomic pairs, with parentheses showing the range used for the calculation of the coordination values. Constrained values are given in bold.

	Coordination number, CNij				
	Si-O (*r*_1_:1.30-*r*_2_:1.90)	B-O (*r*_1_:1.20-*r*_2_:1.75)	U-O (*r*_1_:1.60-*r*_2_:2.70)	Si-Si (*r*_1_:2.80-*r*_2_:3.40)	Si-B (*r*_1_:2.50-*r*_2_:3.40)
REF ^9^	3.70 ± 0.02	3.05 ± 0.02	-	-	-
MUCe	**3.87**	3.40 ± 0.03	**6.60**	1.72 ± 0.1	0.66 ± 0.02
MUNd	**3.86**	3.51 ± 0.03	**6.19**	1.79 ± 0.1	0.67 ± 0.02
MUEu	**3.63**	3.25 ± 0.02	**6.60**	1.65 ± 0.5	0.64 ± 0.02
MUCNE	**3.84**	3.47 ± 0.03	**6.54**	1.61 ± 0.5	0.61 ± 0.02

**Fig. 5:**
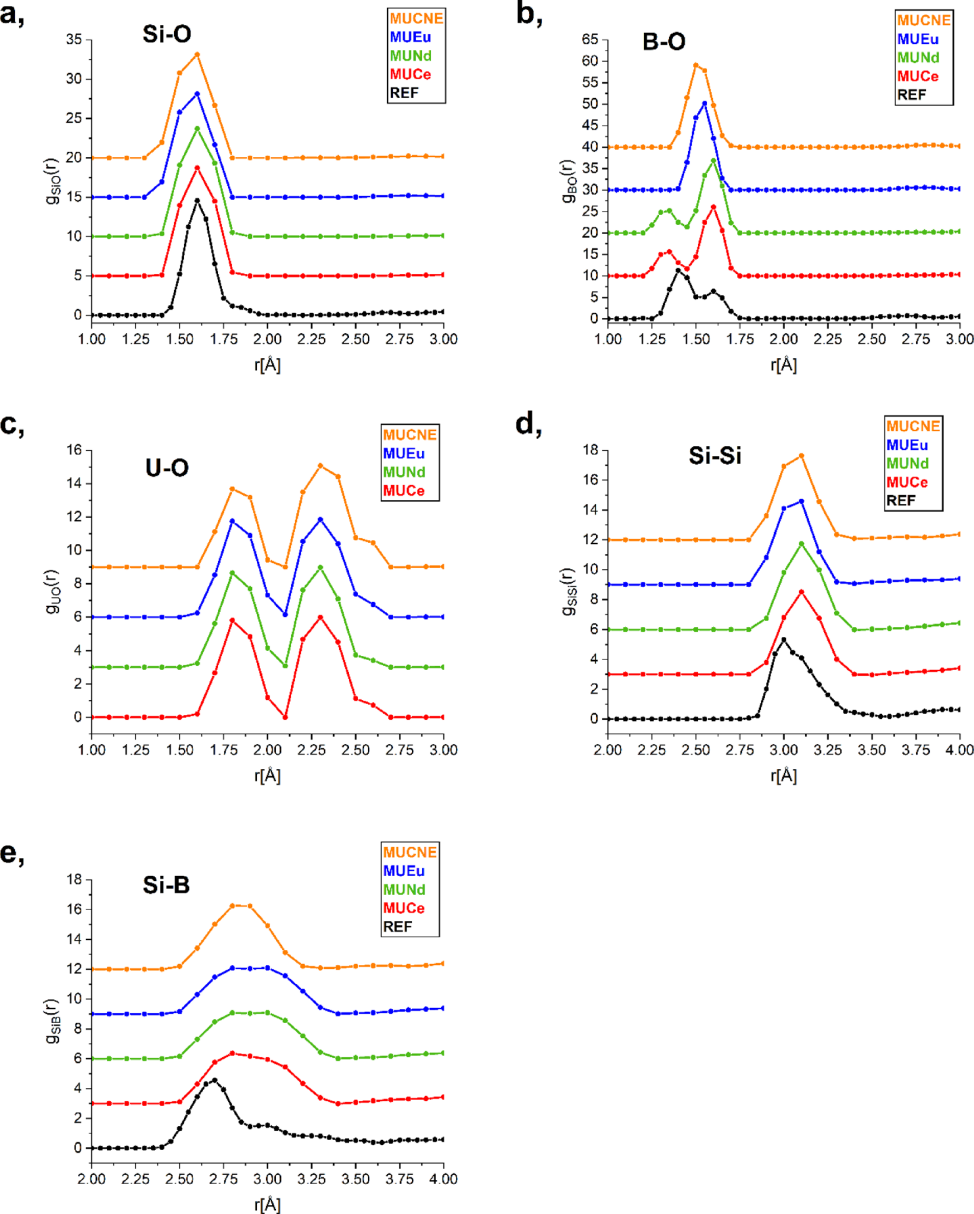
Partial atomic pair correlation functions for Si-O (**a**), B-O (**b**), U-O (**c**), Si-Si (**d**), and Si-B (**e**) with the curves vertically shifted along the Y-axis for clarity.

The Si-O interatomic distance was obtained around 1.60 ± 0.02 Å across all compositions as shown in Fig. 5.

This value is close to the Si-O bond length of the REF glass and also agrees with the literature, where rare earth metals are similarly used as loading ions^44,45^. In contrast, a study involving trivalent lanthanide ions, where sodium silicate glass was loaded with 6 mol% La_2_O_3_ and Dy_2_O_3_, reported bond distances that were 0.04 Å longer^46^. The values for the Si-O coordination number, determined under the specified constraints, are presented in Table 9.

Due to the Si-O coordination constraints the vast majority of Si atoms are surrounded by 4 O neighbors. A consequence of the presence of well-defined SiO_4_ units is that the first peak of g_SiSi_(r) is also well defined. However, a slight deviation from the ideal configuration is noted. The Si-O coordination numbers remain highly similar regarding the MUCe, MUNd and MUCNE samples, while MUEu has the lowest value. A loading of 10 wt% Eu_2_O_3_ with the combination of 20 wt% UO_3_ resulted in a higher concentration of non-bridging oxygen compared to the reference sample. In contrast, the 5.42 wt% Eu_2_O_3_ loading in the glass sample labeled as CJ_1_-Eu, as reported in^47^ did not yield the same increase in non-bridging oxygen. The presence of Ce^III^ and Nd^III^ ions results in the same decrease in the number of non-bridging oxygen atoms, as indicated by the increase in the coordination number compared to the REF glass.

A notable similarity in Si-Si first neighbor distances is observed across the sample series and a small increase is noted relative to the REF glass. For the MUCe and MUNd samples, a value of 3.10 ± 0.05 Å is observed, whereas a slightly lower values of 3.06 ± 0.05 Å and 3.07 ± 0.05 Å are obtained for the MUEu and MUCNE samples. The interatomic distances are somewhat shorter than in the L_2_O_3_-Na_2_O-SiO_2_ glass^48^ and are in good agreement with the values measured in the vitreous SiO_2_, and 70SiO_2_−30Na_2_O glasses^27,49^. Compact SiO_4_ units appear to form in the glass structure, as suggested by both the interatomic distances and coordination numbers.

Regarding the B-O network, where the working wavelength of 0.726 Å allowed a *Q*-range extending to 15.3 Å^−1^, two interatomic distances revealed two distinct peaks with a notable decreasing trend for the first peak as shown in Table 8; Fig. 5. In contrast, the MUEu and MUCNE samples exhibited a broader distribution with a single peak. This is due to the medium *Q*-range in the neutron diffraction measurements (0.45–9.8 Å^−1^), thus the limited resolution of the PSD instrument the B-O peaks for MUEu and MUCNE are not resolved and have a non physical split shape. Our previous studies indicate that the intensity of the peak at a shorter distance (~ 1.35 Å) increases when Ln ions are incorporated into the glass structure. There is no significant difference in peak intensities between Ce, Nd, and Eu loading^29^. However, concerning U loading, we observed that increasing the UO_3_ content affects the relative intensities of the two peaks. As the UO_3_ content increases, the intensity of the first peak increases, and by 40 wt%, it surpasses the peak at 1.60 Å^8^. As a result, the peak symmetry for MUCe and MUNd samples are identical due to the equal Ln and U content. The broader peak positions for the MUEu and MUCNE samples show strong agreement, although the measured bond lengths are slightly longer than those reported in^47,50^. For a more detailed understanding of the B-O connections, a coordination number distribution analysis was carried out, as the ratio of BO_3_ and BO_4_ units is crucial for the formation of the basic matrix glass structure. Uranium trioxide and Ln oxides behave differently: while the addition of U leads to a decrease in four-coordinated B, acting as an intermediate oxide according to literature^8,51^ Ln ions act as modifiers^47,52,53^. As their concentration increases, the B-O coordination number shifts from 3 to 4. This is reflected in the increase in coordination number, attributed to the higher number of [BO_4_]^−^ units, thus reducing the non-bridging oxygens and promoting isomerization: BO_3_ + O^−^ → [BO_4_]. These results are consistent with our previous study^7^showing that when the same amounts of Ln ions are incorporated into the basic glass structure, the coordination numbers remain quite similar.

The Si-B correlation is observed over a broad range between 2.4 ± 0.05 Å and 3.4 ± 0.05 Å, indicating an interaction between Si-centered and B-centered groups^54,55^. These results suggest that the short-range order is composed of correlating tetrahedral SiO_4_, trigonal BO_3_, and tetrahedral BO_4_ units, which establish a basic network structure through mixed ^4^Si-O-^3^B and^4^Si-O-^4^B linkages^54,56^.

**Fig. 6:**
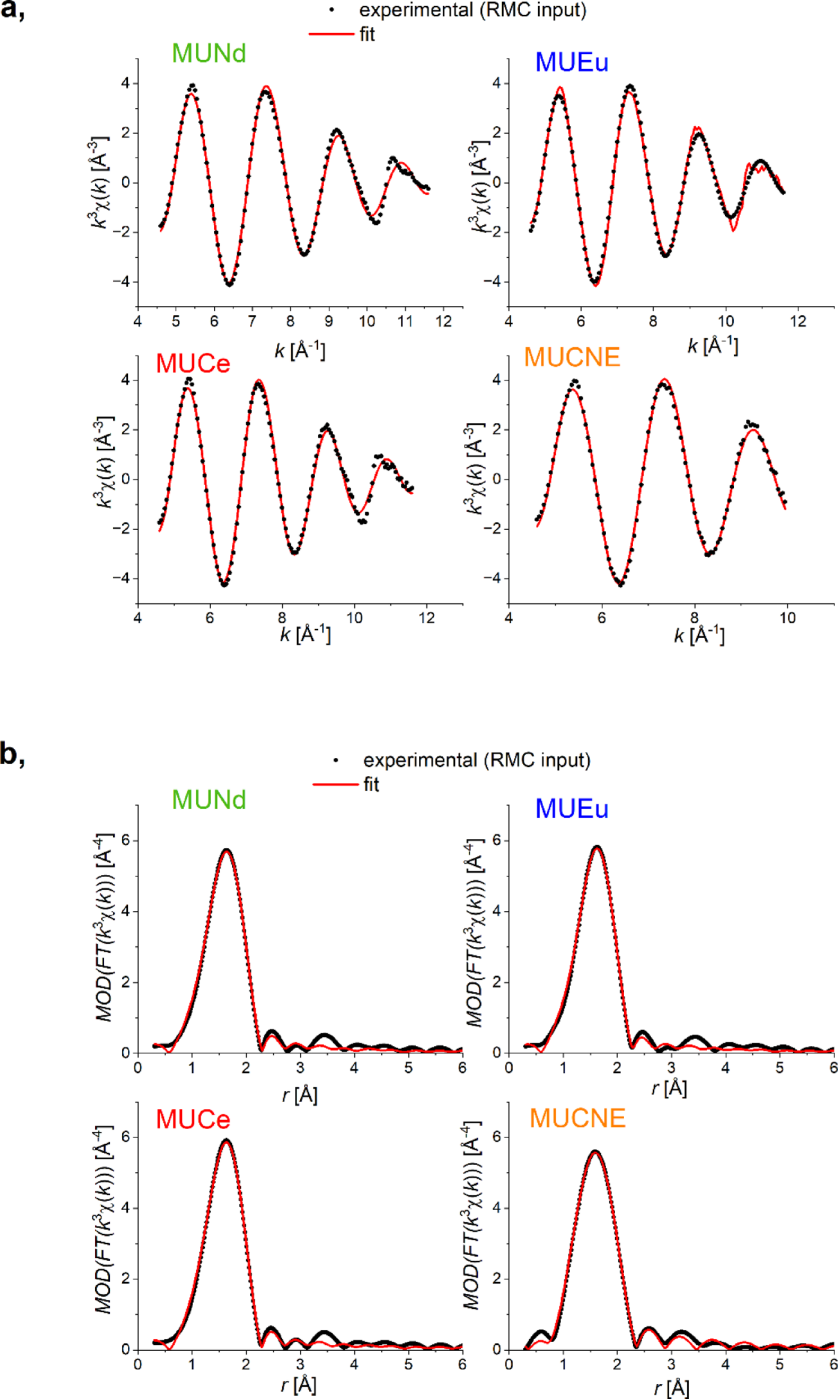
EXAFS data fitting for the U-O partial pair correlation function using the U L_III_-edge: fitting in k-space (**a**) and fitting in r-space (**b**).

The U-O partial atomic pair correlation functions show two characteristic distances at about 1.83 ± 0.02 Å and 2.30 ± 0.04 Å in all samples as shown in Fig. 5; Table 8. Furthermore, the fitting of the EXAFS data used for the U-O partial pair correlation function in both k-space and r-space is presented in Fig. 6.

These values are consistent with earlier findings in a glass containing 30 wt% UO_3_^8^. The results of the RMC simulation are also consistent with the direct analysis of the U L_III_ EXAFS spectra (see below). The average U-O coordination numbers corresponding to the shorter and longer U-O distances are ~ 2 and ~ 4, respectively. It is reasonable to assume that the shorter distance belongs to axially positioned O atoms, while the O neighbors at around 2.29 Å are in an equatorial arrangement^3,57–59^. We note here that curve fitting and RMC modeling give remarkably similar results: there is a shorter U-O peak at ~ 1.83 Å and a longer, composite peak with a center of mass close to 2.3 Å. Though EXAFS spectra themselves do not clarify the very nature of this composite peak, XANES analyses reveal that it consists of U^VI^-O_equatorial_ and U^V^-O distances.

### X-ray photoelectron spectroscopy of the loaded glass surfaces

**Fig. 7:**
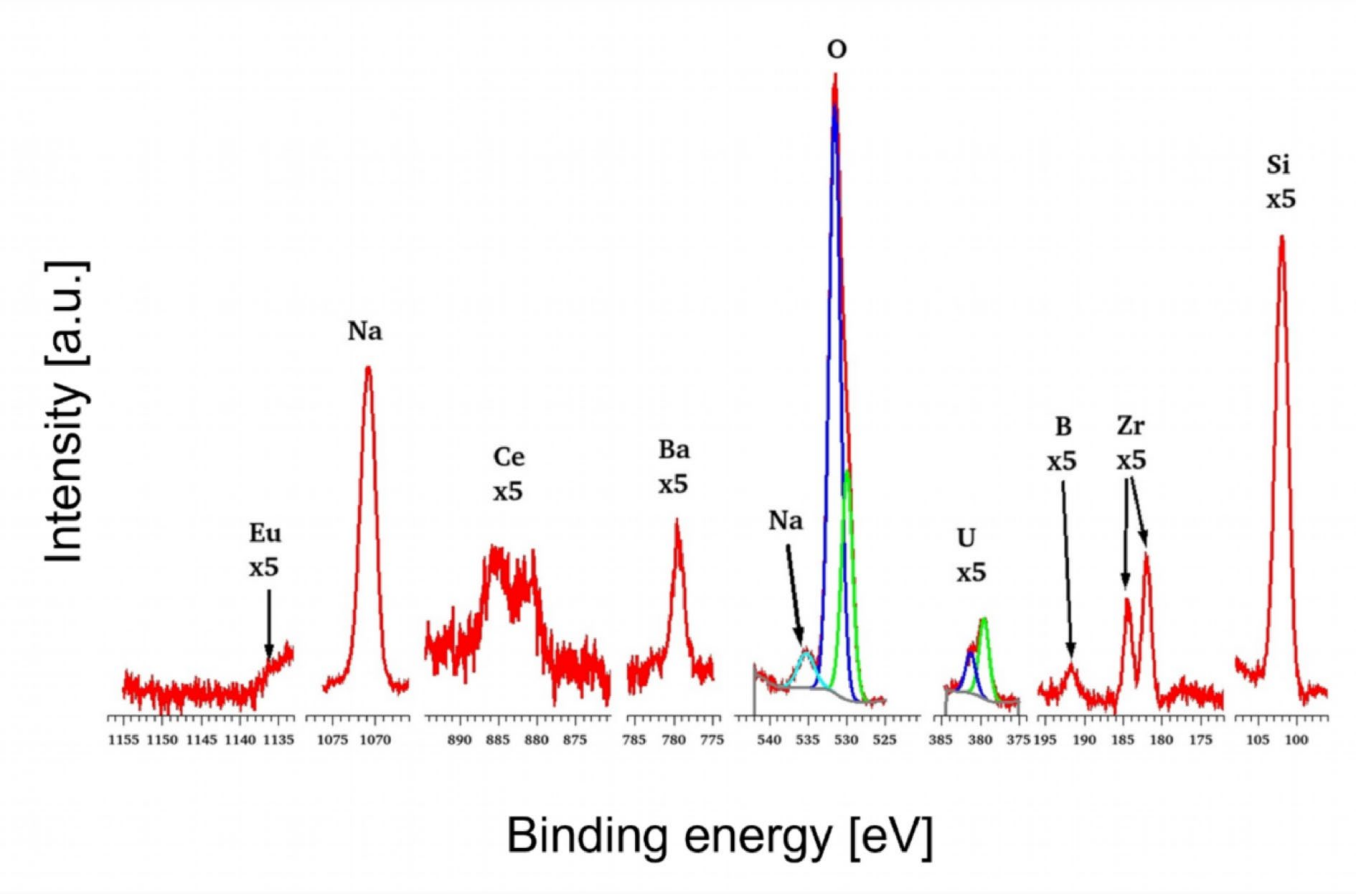
XPS spectrum of sample MUCNE, the carbon peak is omitted since it is not part of the basic glass structure and Nd is also excluded due to the challenges presented by oxygen loss peak.

XPS spectra for all elements were generally well detected, with the exception of Nd. Unfortunately, the highest intensity Nd 3 d peaks are located near the O Auger peaks, specifically in the region where O loss peaks are prominent as shown in Figure S3 in Supplementary material. Due to the high O content in these glass materials, which produces strong and significant loss peaks, the low concentration of Nd could not be detected in this region. Additional attempts to detect Nd using the Nd 4 d peak (120 eV) and Nd 3p peak (1298 eV) were also proved unsuccessful due to their lower intensities. One of the XPS spectra of sample MUCNE are shown below in Fig. 7. The detected peaks are decomposed into sub-components where it is meaningful. For oxygen, the metallic oxides (green on the graph) can be distinguished from the non-metallic oxides (blue on the graph), such as SiO_2_. Additionally, a Na Auger peak is visible near the O peak (turquoise on the graph). As for U, the 4f 7/2 peak presents a double peak structure, reflecting the separation between U^IV^ (green) and U^VI^ (blue) and also including an intermediate state U^V^ (turquoise) between them.

The calculated surface concentrations are averaged over the three positions and the average values are shown in Table 10. All details are provided in Table S1 in Supplementary material.

**Table 10:** Average surface concentrations for the glassy samples obtained by XPS. Nd is not determined due to measurement limitations as discussed in the text.

	Average surface concentrations [at%]											
	Si	Na	O	Zr	B	U^IV^	U^V^	U^VI^	Ba	Ce	Eu	Nd
MUCe	20.4	14.6	61.2	0.4	2.9	0.08	0.03	0.12	0.18	0.11		n.d.
MUNd	15.5	11.9	70.9	0.1	1.4	0.05	0.03	0.05	0.07			n.d.
MUEu	19.9	12.2	61.4	0.6	5.6	0.06		0.11	0.11		0.1	n.d.
MUCNE	22.0	9.4	63.6	0.8	3.4	0.03	0.05	0.06	0.22	0.39	0.1	n.d.

**Fig. 8:**
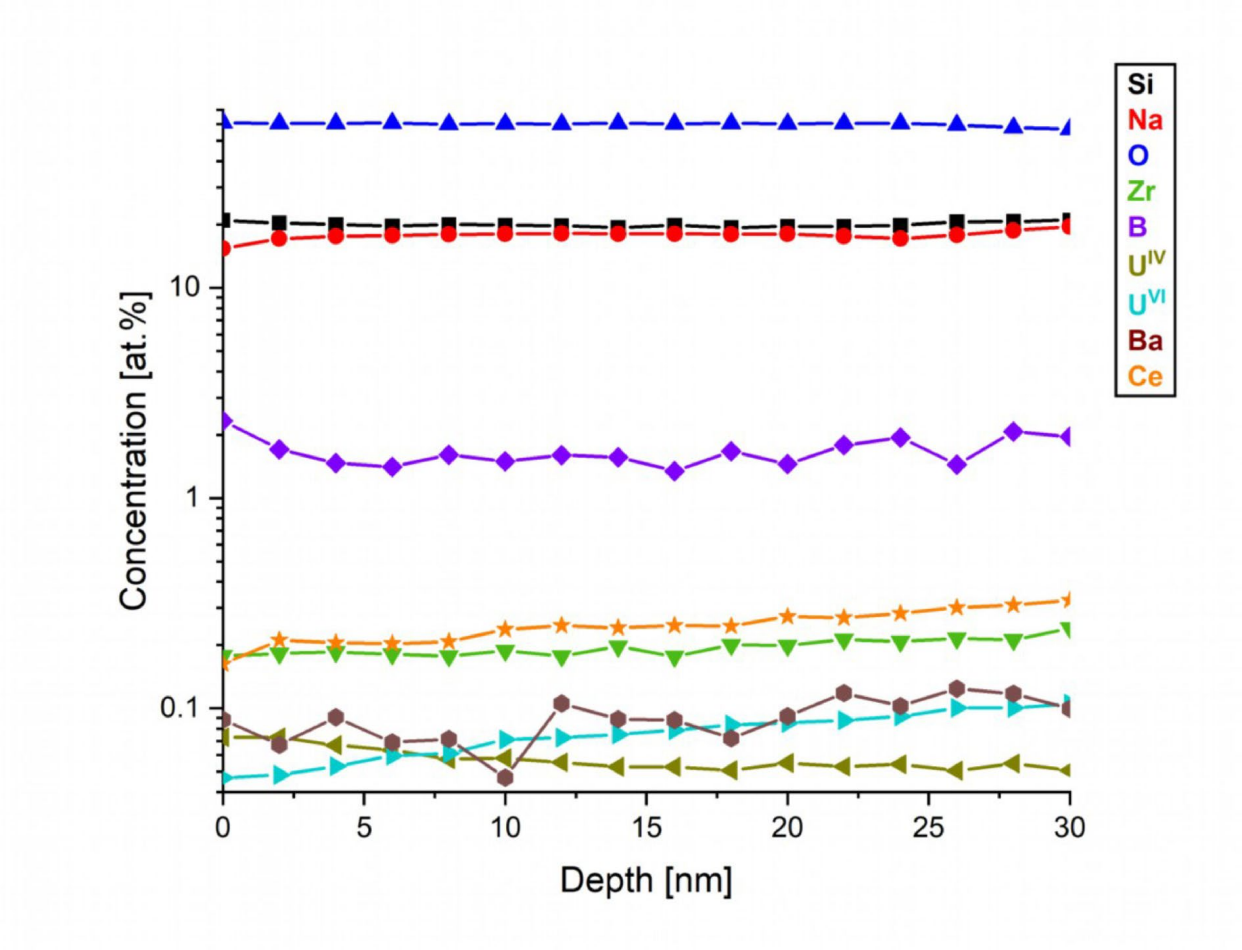
XPS depth profile of the MUCNE sample surface. For the visibility of low concentration components, the concentration axis is shown in logarithmic scale.

The decomposition of the O peak is uncertain, given the small energy difference (1.6 eV) between metallic and non-metallic oxides, which is smaller than the own width of components (2 eV), so O subcomponents were not detailed in Table 10. However, the decomposition of U 4f peak is reliable since the splitting of U^IV^ and U^VI^ components is high enough (3.4 eV). The total O values carry some error, as surface contamination caused over-detection by approximately 10% on original surfaces. The Eu 3 d 5/2 peak was only partially detected, the total quantity was estimated by extrapolation, thus making it less precise. Eu is present in the Eu^III^ state, consistent with the normalized XANES spectra results. Ce in the Ce^III^ state, U in mixed U^IV^, U^V^ and U^VI^ states, however, these results differ slightly from those obtained from the XANES results. In the case of Ce, the ultra-high vacuum conditions during the XPS measurements, combined with X-ray radiation, significantly promote its reduction, leading to an overestimation of the Ce^III^ concentration. This effect is particularly pronounced in the Ce 3 d peaks, which explains why the lower Ce^IV^ ratio observed in the XANES results cannot be reproduced in XPS measurements^60,61^. A similar effect is observed for U, which explains the detection of U^IV^ in small concentrations^62^. B is present as B^III^, and Zr as Zr^IV^. The U^V^ fraction of U in case of MUEu is not available, as the U peak is too small and noisy to allow for precise decomposition. While U is detected at very low concentrations (~ 0.1 at%), this should not be interpreted as an absence of U^V^, but rather as a limitation in decomposition reliability at this concentration^63^. For samples MUEu and MUCNE, the depth distributions were measured, the latter is shown in Fig. 8.

The concentration dependence show rather stable values with little alteration with depth. There is some B segregation to surface which results in B decreasing with depth. The concentrations of dopants such as Zr, Ce and Ba are weakened at the surface and, thus, increase as depth increases. The U^IV^/U^VI^ ratio appears to follow a distinctive profile, overall, the total amount of U seems to rise with depth. A similar trend was reported by Praveena et al., who observed a decreasing contribution of U^IV^ relative to U^VI^ with increasing uranium loading in borosilicate glass matrices^33^. In this study, U^IV^ was clearly present at 2 mol% UO_3_, but its proportion diminished at 4 and 6 mol% loadings. In our sample, which contains approximately 3.4 mol% UO_3,_ the U^IV^/U^VI^ ratio also appears to vary with depth, suggesting that both uranium content and local conditions influence the oxidation state distribution.

### Leaching test results

Composition of the leachates obtained by ICP-OES, together with the results for the REF glass and the initial SBPW solution are provided in Table 11.

**Table 11:** ICP-OES results for the leachates, along with previously obtained data for REF glass and SBPW^8^. Zr concentrations are not tabulated as they remained below the detection limit for all samples.

	Concentration, c_i_ [mg/L]							
	Si	B	Na	Ba	U	Ce	Nd	Eu
SBPW	5.90	0.120	492	-	-	-	-	-
					-	-	-	-
REF-3D	152	44.5	780	0.501	-	-	-	-
REF-7D	335	76.9	990	0.587	-	-	-	-
REF-10D	397	82.3	1120	0.556	-	-	-	-
MUCe-3D	291	41.4	1150	0.195	0.0230	< LOD	-	-
MUCe-7D	1010	114	2080	0.0580	0.282	4.34E-03	-	-
MUCe-10D	1490	167	2890	0.0360	0.534	2.96E-03	-	-
MUNd-3D	1140	189	2200	0.0900	0.441	-	2.47E-03	-
MUNd-7D	2030	387	3930	0.313	1.36	-	4.65E-03	-
MUNd-10D	4000	557	5650	0.164	3.59	-	8.03E-03	-
MUEu-3D	21.0	23.5	626	0.619	0.0210	-	-	1.71E-04
MUEu-7D	2370	195	3410	0.101	0.397	-	-	1.82E-03
MUEu-10D	2750	254	4850	0.0740	0.414	-	-	< LOD
MUCNE-3D	24.3	6.33	589	0.681	0.0120	2.53E-03	< LOD	3.55E-04
MUCNE-7D	21.9	7.59	675	0.267	0.0100	3.20E-03	< LOD	< LOD
MUCNE-10D	19.6	8.41	692	0.226	0.0140	7.41E-04	< LOD	1.46E-04

In comparison to the initial SBPW solution, elevated concentrations of Si, B, and Na were observed across all samples, which can be attributed to glass dissolution. For each sample, these measured values increased over time, with the highest concentrations found in the MUNd sample. By the 10th day, the MUCe and MUNd samples showed Si and B values that were an order of magnitude higher than those of the REF-10D sample. Although the concentration of Na remained within the same order of magnitude, it was at least twice as high in all samples. In the case of MUCNE, significantly lower concentrations were measured compared to the REF glass, not only on the final day but also at every sampling time, with Si, B, and Na levels consistently lower than those in the REF glass. The concentration of Ba decreases over time, a noticeable drop is observed by day 10 in both MUCe and MUEu samples, the Zr component of the basic glass is below the detection limit in the leachates. (The estimated detection limit of ICP-OES is 0.0007 mg/L in axial view.) Table 12 contains the normalized concentrations, mass loss, and glass dissolution rates for Si, B, U, Ce, Nd, and Eu in the lanthanide and uranium loaded borosilicate glass samples. During the PCT test, a stable borosilicate glass system is expected to keep the normalized release of glass forming elements such as Si and B below 2 g/m^2^/day, which was achieved by all the samples in the series^30^. Given its high solubility in aqueous solutions, B serves as an excellent marker for assessing the glass reaction with the leaching solution. For the MUCe, MUNd, and MUEu samples, an increasing trend in the *NR* values for boron is observed over time, with consistently higher values at each point compared to the REF glass. Compared to our previous work on the REF glass loaded with varying content of UO_3_, notable differences can be observed. When only U is incorporated into the system, the average glass dissolution rate is in the range of hundredths. However, with the presence of both U and Ln, the dissolution rate typically increases to the range of tenth^8^. A notable difference is the increasing trend in *NR* values. Typically, dissolution is reduced by the formation of a surface layer that prevents further glass degradation^64,65^. This phenomenon was observed in both Ln and U loaded glass matrices. However, when both elements are present at the same time, this layer did not form within the experiment’s time frame. Based on our previous measurements, the combined presence of U and Ln seems to weaken the glass structure. A decreasing trend was evident within 10 days for samples loaded with 30 wt% lanthanides, whereas this trend has not been observed for samples with 20 wt% U and 10 wt% lanthanides On the other hand, the MUCNE sample displays both an expected decreasing trend and the lowest *NR* values observed in the study. Comparing the uranium *NR* values with those from earlier U loaded glass results, it is noticeable that they are generally within the same order of magnitude, except for the MUNd sample, which shows an order of 10^−3^ by the 10th day. The *NC* values of U are in good agreement with the results obtained in literature^66^where lower values were also measured for U than for the glass forming elements. When comparing the *NR* values of the surrogates with our previous lanthanide loaded glass results, we find that the values remain within the same order of magnitude, with the highest values still observed in the MUNd sample. A comparison of the *NC* values for Ce and Nd with data published in literature^67^ reveals that the MUCe, MUNd, and MUCNE samples exhibit values an order of magnitude higher, with the exception of the 10th day result for the MUCNE sample, which falls within the same order of magnitude. The overall data suggest that the MUNd sample produces a glass with a generally weaker structure, despite containing only 60 wt% glass matrix, the MUCNE sample shows the most stable structure during leaching, outperforming the samples with higher glass matrix content.

**Table 12:** Normalized concentrations, mass loss, and glass dissolution rates for si, B, U, ce, nd, and Eu in the lanthanide and uranium loaded borosilicate glass sample series were evaluated during the PCT-B test from days 3 to 10.

	REF 3D	REF 7D	REF 10D	MUCe 3D	MUCe 7D	MUCe 10D	MUNd 3D	MUNd 7D	MUNd 10D	MUEu 3D	MUEu 7D	MUEu 10D	MUCNE 3D	MUCNE 7D	MUCNE 10D
*NC*(Si) [g/L]	0.686	1.516	1.787	1.863	6.491	9.536	7.292	13.03	25.67	0.135	15.17	17.60	0.182	0.164	0.146
*f*(Si) -	0.222	0.222	0.222	0.156	0.156	0.156	0.156	0.156	0.156	0.156	0.156	0.156	0.134	0.134	0.134
*NL*(Si) [g/m^2^]	0.43	0.94	1.11	1.15	4.06	5.94	4.535	8.129	16.08	0.084	9.446	10.97	0.113	0.102	0.091
*NR*(Si) [g/(m^2^d)]	0.142	0.134	0.111	0.385	0.580	0.594	1.512	1.161	1.608	0.028	1.349	1.097	0.038	0.015	0.009
*NC*(B) [g/L]	1.400	2.424	2.592	1.897	5.220	7.628	8.679	17.73	25.51	1.078	8.938	11.64	0.339	0.405	0.449
*f*(B) -	0.032	0.032	0.032	0.022	0.022	0.022	0.022	0.022	0.022	0.022	0.022	0.022	0.019	0.019	0.019
*NL*(B) [g/m^2^]	0.87	1.50	1.61	1.176	3.264	4.755	5.398	11.06	15.98	0.671	5.57	7.25	0.210	0.252	0.279
*NR*(B) [g/(m^2^d)]	0.29	0.214	0.161	0.392	0.466	0.475	1.799	1.580	1.598	0.224	0.795	0.725	0.070	0.036	0.028
*NC*(U) [g/L]	-	-	-	1.41E-04	1.70E-03	3.21E-03	2.65E-03	8.18E-03	2.16E-02	1.29E-04	2.39E-03	2.49E-03	1.47E-04	1.23E-04	1.72E-04
*f*(U) -	-	-	-	0.166	0.166	0.166	0.166	0.166	0.166	0.166	0.166	0.166	0.083	0.083	0.083
*NL*(U) [g/m^2^]	-	-	-	8.76E-05	1.06E-03	2.00E-03	1.65E-03	5.10E-03	1.35E-02	8.03E-05	1.49E-03	1.55E-03	9.16E-05	7.65E-05	1.07E-04
*NR*(U) [g/(m^2^d)]	-	-	-	2.92E-05	1.51E-04	2.00E-04	5.50E-04	7.29E-04	1.35E-03	2.68E-05	2.13E-04	1.55E-04	3.05E-05	1.09E-05	1.07E-05
*NC*(Ce) [g/L]	-	-	-	< LOD	5.33E-05	3.64E-05	-	-	-	-	-	-	3.11E-05	3.93E-05	9.10E-06
*f*(Ce) -	-	-	-	0.081	0.081	0.081	-	-	-	-	-	-	0.081	0.081	0.081
*NL*(Ce) [g/m^2^]	-	-	-	-	3.33E-05	2.27E-05	-	-	-	-	-	-	1.93E-05	2.45E-05	5.65E-06
*NR*(Ce) [g/(m^2^d)]	-	-	-	-	4.76E-06	2.27E-06	-	-	-	-	-	-			
*NC*(Nd) [g/L]	-	-	-	-	-	-	2.88E-05	5.42E-05	9.37E-05	-	-	-	< LOD	< LOD	< LOD
*f*(Nd) -	-	-	-	-	-	-	0.086	0.086	0.086	-	-	-	0.086	0.086	0.086
*NL*(Nd) [g/m^2^]	-	-	-	-	-	-	1.79E-05	3.38E-05	5.87E-05	-	-	-	-	-	-
*NR*(Nd) [g/(m^2^d)]	-	-	-	-	-	-	5.97E-06	4.83E-06	5.87E-06	-	-	-	-	-	-
*NC*(Eu) [g/L]	-	-	-	-	-	-	-	-	-	1.98E-06	2.11E-05	< LOD	4.11E-06	< LOD	1.69E-06
*f*(Eu) -	-	-	-	-	-	-	-	-	-	0.086	0.086	0.086	0.086	0.086	0.086
*NL*(Eu) [g/m^2^]	-	-	-	-	-	-	-	-	-	1.23E-06	1.31E-05	-	2.55E-06	-	1.05E-06
*NR*(Eu) [g/(m^2^d)]	-	-	-	-	-	-	-	-	-	4.11E-07	1.87E-06	-	8.51E-07	-	1.05E-07

## Conclusion

Borosilicate glass matrix samples with varying compositions: 70 wt% [Matrix] combined with 20 wt% UO_3_ and 10 wt% CeO_2_; 10 wt% Nd_2_O_3_; and 10 wt% Eu_2_O_3_; and a composition of 60 wt% [Matrix] with 10 wt% UO_3_, 10 wt% CeO_2_, 10 wt% Nd_2_O_3_, and 10 wt% Eu_2_O_3_ were synthesized and analyzed using X-ray absorption techniques, neutron diffraction coupled with RMC, and X-ray photoelectron spectroscopy were used to investigate the effects of lanthanide-uranium incorporation on the local atomic environment in the borosilicate glass. Additionally, a product consistency test was performed to assess the chemical durability of the glass specimens immersed in porewater. The U L_III_-edge spectra were modeled with U^VI^ ions as uranyl in a linear (O = U = O) arrangement with six equatorial O atoms, and U^V^ in octahedral coordination. Notably, compared to the previous U containing glass samples, there was a slight increase in the U^V^-O bond length by 0.05–0.07 Å, along with increased static disorder of the U^V^O_5_ octahedra. Additionally, the structural function of Nd in the glasses is not influenced by the addition of U, Ce, or Eu. In the MUCe sample, LCF analysis confirmed 3.9% Ce^IV^ content, while the MUCNE sample showed a notable Ce^IV^ fraction (~ 12.3%). No redox sensitivity was observed, with Nd in the MUNd and MUCNE samples existing purely in the Nd^III^ oxidation state. Similarly, both samples containing Eu exhibited only Eu^III^ ions. LCF analysis of the U L_III_-edge indicated no U^IV^ presence, consistently showing U^VI^ and U^V^ in similar proportions. Neutron diffraction combined with Reverse Monte Carlo simulations confirmed that the basic structure of the mixed glass closely resembles that of the REF glass, composed of tetrahedral SiO_4_ and mixed BO_3_/BO_4_ units. Like the U containing samples, the incorporation of both U and Ln resulted in no significant structural changes compared to the REF glass. A coordination number distribution analysis was performed to further understand B-O bonding, given the critical role of the BO_3_ and BO_4_ ratio in forming the basic structure of the glass matrix. While U acts as an intermediate oxide and reduces the number of four-coordinated B, Ln ions serve as modifiers, with their increasing concentration shifting the B-O coordination from 3 to 4. These findings showed that adding equal amounts of Ln ions to the basic glass structure yields similar coordination numbers. The samples show a reduction in non-bridging oxygens and promoting isomerization: BO_3_ + O⁻ → [BO_4_]⁻.

XPS depth analysis reveals that B concentration decreases with increasing depth, whereas the concentrations of dopants like Zr, Ce, and Ba increase. The U^IV^/U^VI^ ratio also follows a distinct pattern, with the overall U content appearing to increase as depth increases. Compared to the initial SBPW solution, the PCT test results showed increased concentrations of Si, B, and Na in all samples, indicating glass dissolution. These concentrations increased over time in each sample, with the highest levels observed in the MUNd sample.

Overall, the data suggests that the MUNd sample has a relatively weaker glass structure, while the MUCNE sample, despite containing higher, 40 wt% simulated waste components, exhibited the most stable structure during leaching, outperforming samples with higher glass matrix content.

The studied borosilicate glass matrix shows a strong capacity to incorporate high concentrations of actinides and uranium, suggesting its viability for use in high-level nuclear waste disposal.

## Supplementary Information

Below is the link to the electronic supplementary material.

Supplementary Material 1

## Data Availability

Datasets generated during the current study are available from the corresponding author (fabian.margit@ek.hun-ren.hu) on reasonable request.

## Declarations

### Supplementary Information

Supplementary material available, calculated surface concentrations, variations in the % of U^IV^ relative to the atomic number of the lanthanides, the region of Nd peak detected on the MUNd sample.
