# Supplementary material for: Structural characterization of uranium and lanthanide loaded borosilicate glass matrix
Source: Sci Rep. 2025 Aug 4;15:28352. doi: 10.1038/s41598-025-13166-1 (PMC12322290; doi:10.1038/s41598-025-13166-1)
Supplement: Supplementary file 1 — Supplementary Material 1 [file 41598_2025_13166_MOESM1_ESM.docx]

**Structural characterization of uranium and lanthanide loaded borosilicate glass matrix**

I. Tolnai^1^, J. Osan^1^, P. Jovari^2^, F. Pinakidou^3^, A. Sulyok^1^, M. Fabian^1*^

*^1^HUN-REN Centre for Energy Research, Konkoly Thege St. 29-33., Budapest, 1121, Hungary*

*^2^HUN-REN Wigner Research Centre for Physics, Konkoly Thege St. 29-33., Budapest, 1121, Hungary*

*^3^Aristotle University of Thessaloniki, School of Physics, Thessaloniki, 54124, Greece*

*Corresponding Author: [*fabian.margit@ek.hun-ren.hu*](mailto:fabian.margit@ek.hun-ren.hu), ORCID: 0000-0002-6528-4695

Table S1 The calculated surface concentrations are averaged across the three positions for the glassy specimens. The measurement of MUNd P1 seems out of order thus it was omitted in the average calculation.

|  | Calculated surface concentrations [at.%] | | | | | | | | | | |
| --- | --- | --- | --- | --- | --- | --- | --- | --- | --- | --- | --- |
|  | Si | Na | O | Zr | B | U^IV^ | U^V^ | U^VI^ | Ba | Ce | Eu |
| MUCe P1 | 22.4 | 11.9 | 61.2 | 0.49 | 3.39 | 0.09 | 0.03 | 0.11 | 0.17 | 0.15 |  |
| MUCe P2 | 19.8 | 15.5 | 61.1 | 0.42 | 2.74 | 0.06 | 0.05 | 0.10 | 0.15 | 0.11 |  |
| MUCe P3 | 18.8 | 16.4 | 61.2 | 0.43 | 2.60 | 0.08 | 0.00 | 0.14 | 0.22 | 0.08 |  |
| MUNd P1 | 7.5 | 8.1 | 83.5 | 0.08 | 0.79 | 0.03 | 0.00 | 0.03 | 0.04 |  |  |
| MUNd P2 | 20.8 | 11.5 | 65.2 | 0.12 | 2.13 | 0.05 | 0.07 | 0.06 | 0.00 |  |  |
| MUNd P3 | 18.1 | 16.2 | 64.1 | 0.15 | 1.14 | 0.08 | 0.02 | 0.07 | 0.16 |  |  |
| MUEu P1 | 16.6 | 17.0 | 61.9 | 0.44 | 3.74 | 0.05 |  | 0.09 | 0.15 |  | 0.05 |
| MUEu P2 | 21.8 | 8.7 | 61.0 | 0.56 | 7.48 | 0.07 |  | 0.13 | 0.19 |  | 0.07 |
| MUEu P3 | 21.3 | 11.0 | 61.2 | 0.66 | 5.62 | 0.06 |  | 0.10 | 0.00 |  | 0.09 |
| MUCNE P1 | 22.1 | 9.5 | 63.6 | 0.81 | 3.27 | 0.02 | 0.05 | 0.06 | 0.20 | 0.25 | 0.08 |
| MUCNE P2 | 21.9 | 9.3 | 63.5 | 0.76 | 3.59 | 0.04 | 0.04 | 0.06 | 0.23 | 0.53 | 0.06 |


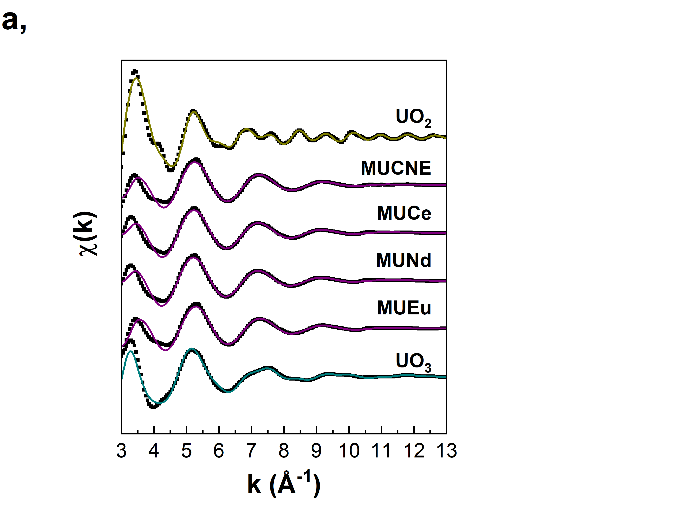

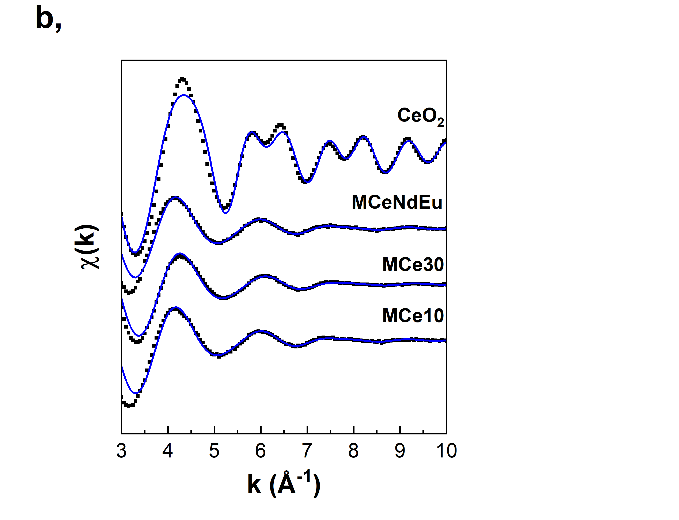


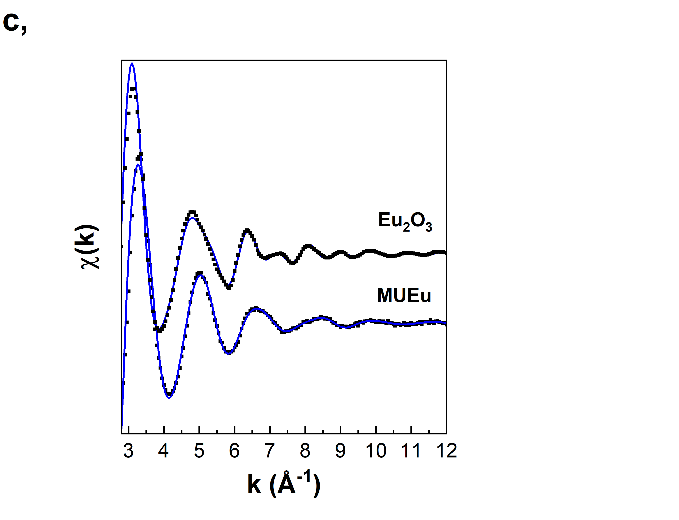

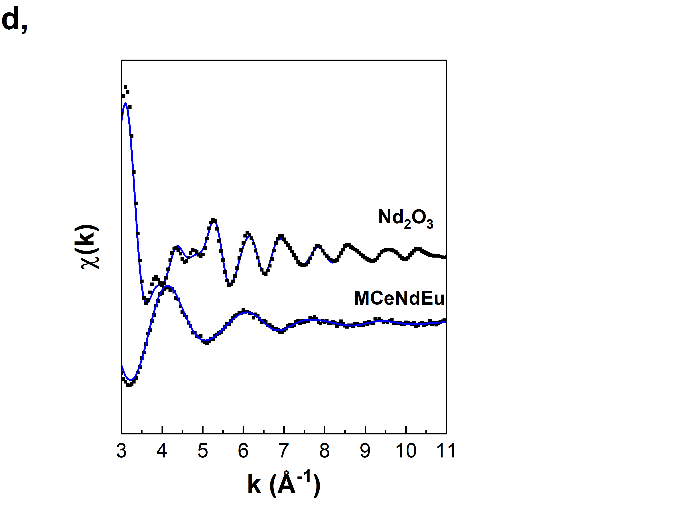


Figure S1 The raw and fitted χ(*k*) EXAFS spectra for the Ln and U loaded glassy samples at the U L_III_-edge (a), for the supplementary Ce loaded samples and MCeNdEu recorded at the Ce K-edge (b), for the MUEu sample at the Eu L_III_-edge (c), and for the MCeNdEu sample at the Nd K-edge (d). The figures includes also the raw and fitted χ(*k*) EXAFS spectra of all reference samples, namely UO_2_, UO_3_, CeO_2_, Eu_2_O_3_, Nd_2_O_3_. The raw data are depicted in square points while the fitting with a solid colored line.


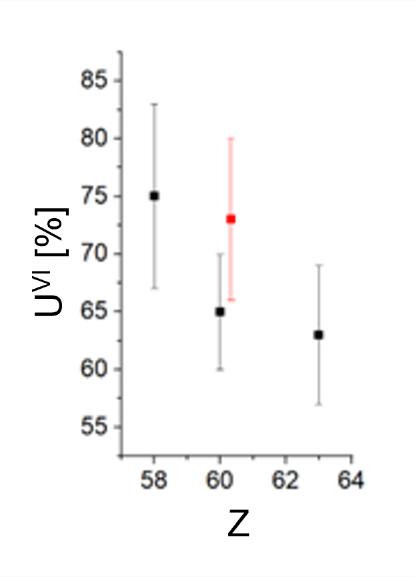


Figure S2 A plot illustrating the variation in the % of U^VI^ relative to the atomic number (*Z*) of the lanthanides. The red point in the graph corresponds to the glass containing all three Ln-oxides, with the average *Z* value used.


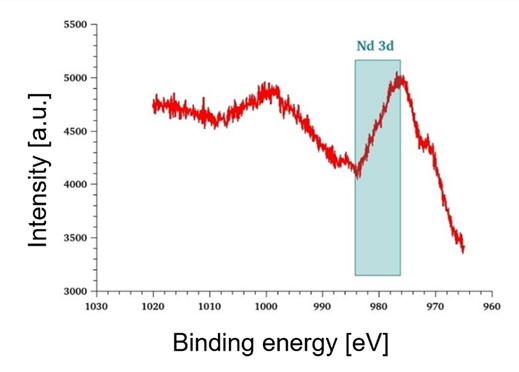


Figure S3 The region of Nd peak detected on the MUNd sample, the possible Nd 3d peak is loaded with wide loss peaks form O Auger peak.
